# **Editorial**: Global excellence in oncology: Latin America 2022

**DOI:** 10.3389/fonc.2022.971225

**Published:** 2022-07-12

**Authors:** Javier Camacho, Carolina Panis

**Affiliations:** ^1^ Department of Pharmacology, Centro de Investigación y de Estudios Avanzados del Instituto Politécnico Nacional, Mexico City, Mexico; ^2^ Laboratory of Tumor Biology, State University of West Paraná – UNIOESTE, Francisco Beltrão, Paraná, Brazil

**Keywords:** Latin America, oncology, global excellence, cancer epidemiology, cancer risk factors

## Introduction

Global action in cancer is urgently needed. Understanding the genetic differences as well as the diverse response to cancer treatments between populations will certainly lead to better cancer diagnosis and therapies. These goals may be achieved mainly *via* international collaborations. Cancer mortality-to-incidence ratio is higher in developing countries in comparison to more developed nations, making such international collaborations crucial.

The present Research Topic *Global Excellence in Oncology: Latin America 2022* aims to show some of the extraordinary scientific efforts of Latin America researchers to contribute to global solutions in cancer. In consequence, the *Research Topic* displays the huge opportunities to work together finding solutions in the benefit of cancer patients. This topic comprises extraordinary works including basic molecular aspects of cancer, exposure to cancer risk-factors, as well as social and economy issues associated to cancer. The contributions include several international collaborations beyond Latin America countries.

In a first article, an elegant multi-institutional international study by Almeida et al. highlights the global nature of this *Research Topic*. The paper results from the teamwork of several Latin America countries and the United States–Latin American Cancer Research Network (US-LACRN) and is the first study in its own. The participants integrated epidemiological profiles influencing breast cancer-overall survival in Latin American patients. The factors studied included socioeconomic, clinical, and molecular features, and contained personal interviews. Interestingly, differences were observed depending on the country of residence, age of the patients, as well as tumor stage at diagnosis and molecular subtype. Definitely, these data will help to identify predictors of breast cancer in populations with diverse sociocultural settings. Besides, as asserted by the authors, this international collaborative work should serve as the basis to reduce breast cancer burden. This statement may be valid not only for Latin American breast cancer patients but more globally.

Next, Llera et al., enriches the *Research Topic* with another very interesting international collaboration studying breast cancer. Once again, the teamwork includes several Latin America countries and US-LACRN. Latin American breast cancer patients are poorly represented in this type of studies worldwide. In this article, the authors show the transcriptomic portrait of locally advanced breast cancer in a cohort of more than 1,000 Latin American patients including the analysis of the potential gene expression-based pathways associated to various tumor subtypes.

The classification-derived risk of recurrence used, helped the authors to improve the discrimination between low- intermediate- and high-risk groups. Besides, by analyzing components of the innated and adaptive immune response, this international collaboration emphasizes the potential benefit that women with aggressive tumors may have if some therapeutic approaches were available for Latin America breast cancer patients. With no doubt, these findings may lead to major analysis by the policy makers responsible for cancer management in some Latin America countries. This study invites to act globally in this issue.

Following with breast cancer studies in this *Research Topic*, a multi-institutional team in Brazil focused on the DNA damage response (DDR) and homologous repair gene alterations in patients exposed to pesticides. Scandolara et al. investigated - in an exceptional manner -alterations in several genes including BRCAs, PALB2 and TP53 which have been associated with several types of cancer. The authors report that patients exposed to pesticides showed more pathogenic tumor variants in comparison with unexposed patients. Remarkably, such tumor variants displayed a higher mutational burden which may be associated with treatment response and clinical outcomes.

It is well documented that pesticide exposure increases cancer risk. Thus, despite that exposure to pesticides should be under strict control, the actual situation may be different especially in developing countries including some Latin America nations. Longer follow-up of patients exposed to pesticide is needed. Nonetheless the contribution of this work should be taken into account by the corresponding regulating offices.

Several Latin America countries have high incidence and mortality rates of melanoma of the skin in comparison to many other developing nations. Then, novel treatments as well as prognostic factors are needed. In a very interesting article of this *Research Topic*, Reichrath et al., describe that low vitamin D levels are associated with poor clinical outcome in advanced melanoma patients treated with BRAF/MEK inhibitors or immunotherapy. They observed that the risk for progressive disease decreases when 25(OH)D levels were increased s.c. Accordingly, overall survival was decreased in patients with severe vitamin-D deficiency. In addition, the adverse events showed a trend to decrease when 25(OH)D was administered. The authors nicely discuss the potential mechanisms involved in the benefit provided by vitamin D.

The current treatments for advanced melanoma are more promising than a few years ago. Nevertheless, because of the high incidence and mortality of melanoma, more interventional studies evaluating the effect of vitamin D are globally deserved. These findings may have relevant clinical implications for advanced melanoma management.

The articles published in this *Research Topic* clearly demonstrate the scientific effort of many Latin America researchers to contribute to global solutions in cancer. This teamwork should help to fight cancer not only in developing countries but worldwide.

We hope these articles to provide the reader an outstanding panorama of the excellence in oncology in Latin America.

## Author contributions

All authors listed have made a substantial, direct, and intellectual contribution to the work and approved it for publication.

## Conflict of Interest

The authors declare that the research was conducted in the absence of any commercial or financial relationships that could be construed as a potential conflict of interest.

## Publisher’s note

All claims expressed in this article are solely those of the authors and do not necessarily represent those of their affiliated organizations, or those of the publisher, the editors and the reviewers. Any product that may be evaluated in this article, or claim that may be made by its manufacturer, is not guaranteed or endorsed by the publisher.

